# The Prenylflavonoid Xanthohumol Reduces Alzheimer-Like Changes and Modulates Multiple Pathogenic Molecular Pathways in the Neuro2a/APP_swe_ Cell Model of AD

**DOI:** 10.3389/fphar.2018.00199

**Published:** 2018-04-04

**Authors:** Xianfeng Huang, Jing Wang, Xiao Chen, Pan Liu, Shujin Wang, Fangchen Song, Zaijun Zhang, Feiqi Zhu, Xinfeng Huang, Jianjun Liu, Guoqiang Song, Peter S. Spencer, Xifei Yang

**Affiliations:** ^1^College of Pharmaceutical Engineering and Life Sciences, Changzhou University, Changzhou, China; ^2^Key Laboratory of Modern Toxicology of Shenzhen, Shenzhen Center for Disease Control and Prevention, Shenzhen, China; ^3^Department of Neurology, The First Hospital of Zibo, Weifang Medical University, Zibo, China; ^4^Key Laboratory of Innovative Chemical Drug Research in Cardio-Cerebrovascular Diseases, Institute of New Drug Research and Guangzhou, College of Pharmacy, Jinan University, Guangzhou, China; ^5^Department of Cognitive Impairment Ward of Neurology, The Third Affiliated Hospital of Shenzhen University, Shenzhen, China; ^6^Department of Neurology, School of Medicine, Oregon Institute of Occupational Health Sciences, Oregon Health and Science University, Portland, OR, United States

**Keywords:** Alzheimer’s disease (AD), xanthohumol (Xn), amyloid-β (Aβ), endoplasmic reticulum (ER) stress, oxidative stress, cytoskeleton

## Abstract

Alzheimer’s disease (AD) is a progressive neurodegenerative disorder that has proved refractory to drug treatment. Given evidence of neuroprotection in animal models of ischemic stroke, we assessed the prenylflavonoid xanthohumol from the Common Hop (*Humulus lupulus* L.) for therapeutic potential in murine neuroblastoma N2a cells stably expressing human Swedish mutant amyloid precursor protein (N2a/APP), a well-characterized cellular model of AD. The ELISA and Western-blot analysis revealed that xanthohumol (Xn) inhibited Aβ accumulation and APP processing, and that Xn ameliorated tau hyperphosphorylation via PP2A, GSK3β pathways in N2a/APP cells. The amelioration of tau hyperphosphorylation by Xn was also validated on HEK293/Tau cells, another cell line with tau hyperphosphorylation. Proteomic analysis (2D-DIGE-coupled MS) revealed a total of 30 differentially expressed lysate proteins in N2a/APP vs. wild-type (WT) N2a cells (N2a/WT), and a total of 21 differentially expressed proteins in lysates of N2a/APP cells in the presence or absence of Xn. Generally, these 51 differential proteins could be classified into seven main categories according to their functions, including: endoplasmic reticulum (ER) stress-associated proteins; oxidative stress-associated proteins; proteasome-associated proteins; ATPase and metabolism-associated proteins; cytoskeleton-associated proteins; molecular chaperones-associated proteins, and others. We used Western-blot analysis to validate Xn-associated changes of some key proteins in several biological/pathogenic processes. Taken together, we show that Xn reduces AD-related changes in stably transfected N2a/APP cells. The underlying mechanisms involve modulation of multiple pathogenic pathways, including those involved in ER stress, oxidative stress, proteasome molecular systems, and the neuronal cytoskeleton. These results suggest Xn may have potential for the treatment of AD and/or neuropathologically related neurodegenerative diseases.

## Introduction

**FIGURE 1:**
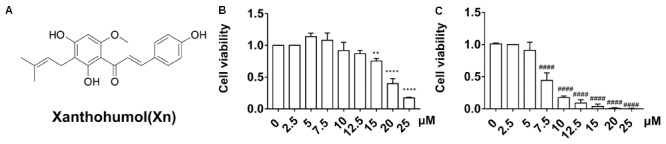
Chemical structure **(A)** and viability of N2a/WT cells **(B)** and N2a/APP cells **(C)** treated for 24 h with 0–25 μM Xn. *N* = 3. ^∗∗^*p* < 0.01, ^∗∗∗∗^*p* < 0.0001 compared with N2a/WT cells treated with vehicle. ^####^*p* < 0.0001 compared with untreated N2a/APP cells.

Xanthohumol (Xn) is the abundant prenylated polyphenol, or chalcone (**Figure 1A**), in cones of the Common Hop (*Humulus lupulus* L.), a species of flowering plant native to temperate regions of the Northern Hemisphere. Xn is reported to scavenge reactive oxygen species (ROS) at relatively low concentrations (Gerhauser et al., 2002; Yamaguchi et al., 2009) while exhibiting pro-oxidative (Strathmann et al., 2010; Blanquer-Rossello et al., 2013) or pro-apoptotic (Strathmann et al., 2010; Festa et al., 2011) effects at higher concentrations. In addition, the Michael Acceptor of this chalcone irreversibly binds to and modifies cysteine residues of proteins, such as those involved in the NF-κB activation pathway, which is inhibited by Harikumar et al. (2009). Both of these chemical properties appear to underwrite the poly-pharmacological activities of Xn, including anti-proliferation (Deeb et al., 2010), anti-inflammation (Dorn et al., 2013), and anti-adipogenesis (Yang et al., 2007).

Xn is also reported to protect rat neuron-like PC12 cells from oxidative damage; to promote neuronal differentiation and neurite outgrowth of mouse embryonic forebrain neural precursors and murine N2a neuroblastoma-derived cells; to improve cognitive flexibility in young mice, and to protect brain tissue in a rodent model of cerebral ischemia (Yen et al., 2012; Oberbauer et al., 2013; Zamzow et al., 2014; Yao et al., 2015). Although the neuroprotective effects of Xn have been attributed to its free-radical scavenging property, the chalcone also positively modulates central regulators of cellular redox and energy balance via actions on endoplasmic reticulum (ER) (activation of nuclear factor E2-related factor 2: Nrf2) and mitochondria (activation of AMP-activated kinase: AMPK) in mouse fibroblasts (Zimmermann et al., 2015).

The aforementioned observations have encouraged the view that prenylflavonoids such as Xn may have therapeutic value in cancer, diabetes, atherosclerosis, acute brain and spinal cord injury and in chronic progressive neurodegenerative disorders by promoting neurogenesis, neuroregeneration, and neuroprotection (Yen et al., 2012; Oberbauer et al., 2013; Zamzow et al., 2014; Yao et al., 2015). In the present study, we have used murine neuroblastoma N2a cells stably transfected with human amyloid precursor protein (APP) to assess whether and how Xn affects molecular mechanisms relevant to Alzheimer’s disease (AD). This is a progressive neurodegenerative disorder in which dysfunction of multiple cellular organelles correlates with the formation of amyloid β (Aβ) plaques and neurofibrillary tangles (NFT) containing hyperphosphorylated tau, the two neuropathological hallmarks of the disease (Ittner and Götz, 2011). We found that Xn attenuated Aβ accumulation and tau phosphorylation via APP processing, GSK-3β and PP2A pathways as well as reducing ER and oxidative stress and related proteasome processing, properties consistent with therapeutic potential in AD and other progressive neurodegenerative disorders with AD-related neuropathology.

## Materials and Methods

### Reagents

Xn (stated ≥98%) was purchased from Aladdin Company Biochemical Technology Co., Ltd. (Shanghai, China). The stock solution of Xn (10 mM) was prepared in dimethylsulfoxide (DMSO, Thermo Fisher Scientific, Waltham, MA, United States) and was used directly. Selections of antibodies are listed in **Table 1**.

**Table 1:** Brands and usages of the primary antibodies.

Antibody	Specificity	Type	Dilution	Source
t-APP	Total APP	mAb	1:3000	Abcam
sAPPα	sAPPα (2B3)	mAb	1:50	Immuno-Biological
BACE1	BACE1	mAb	1:1000	Cell Signaling
PS1	Presenilin 1	mAb	1:1000	Cell Signaling
pS396	Phosphorylated tau at Ser396	pAb	1:20000	Abcam
pS404	Phosphorylated tau at Ser404	pAb	1:3000	Santa Cruz
pT231	Phosphorylated tau atThr231	mAb	1:1000	Thermo Fisher Scientific
pS262	Phosphorylated tau at Ser262	pAb	1:1000	Thermo Fisher Scientific
tau 1	Non-phosphorylated tau Ser195/Ser198/Ser199/Ser202	mAb	1:200000	Millipore
tau 5	Total tau	mAb	1:3000	Abcam
p-GSK3α/β(Ser21/9)	Phosphorylated GSK3α/β at Ser21/9	pAb	1:1000	Cell Signaling
GSK3α/β	Total GSK-3α/β	pAb	1:1000	Cell Signaling
p-PP2A(Y307)	Phosphorylated PP2A at Y307	pAb	1:1000	R&D systems
PP2A	Total PP2A	pAb	1:1000	Cell Signaling
P35/P25	Total P35/25	pAb	1:1000	Cell Signaling
CDK5	Total CDK5	mAb	1:3000	Abcam
GRP78	GRP78(A-10)	mAb	1:1000	Santa Cruz
PDIA1	PDIA1[RL90]	pAb	1:1000	Abcam
PRDX4	Peroxiredoxin 4	pAb	1:1000	Abcam
8-OHdG	8-Oxo-2′-deoxyguanosine	pAb	1:200	Abcam
p-PERK	Phospho-PERK (Thr980) (16F8)	mAb	1:1000	Cell Signaling
p-eIF2α	Phospho-eIF2 α (Ser51) (D9G8)	mAb	1:1000	Cell Signaling
eIF2α	eIF2α (D7D3)	mAb	1:1000	Cell Signaling
ATF-4	ATF-4(D4B8)	mAb	1:1000	Cell Signaling
CHOP	CHOP(L63F7)	mAb	1:1000	Cell Signaling

### Cell and Cell Culture

Wild-type murine neuroblastoma Neuro2a cells (N2a/WT) and N2a stably transfected with human APP Swedish mutant (N2a/APP) were gifts from Professor Jian-Zhi Wang (Tongji Medical School, Wuhan, China). The cells were maintained in medium consisting of an equivalent volume of Dulbecco’s modified Eagle’s medium (DMEM) and Opti-MEM with 5% fetal bovine serum in 5% CO_2_ at 37°C. Stably transfected cells were screened in the presence of 0.2 g/L Genticin (Thermo Fisher Scientific). Human Embryonic Kidney 293 cells stably transfected with tau protein (HEK293/Tau) were also gifted by Prof. Jian-Zhi Wang. The cells were cultivated in DMEM with 5% fetal bovine serum and. 0.2 g/L Genticin in 5% CO_2_ at 37°C. The cells were grown in 25 cm^2^ or 75 cm^2^ culture flasks and passaged when there were 1.2 × 10^6^ cells in a 25 cm^2^ flask and 3–4 × 10^6^ in a 75 cm^2^ flask.

### Cell Viability Assay

N2a/WT or N2a/APP cells were placed on a 96-well cell culture microplate (10^4^ cell per well). When the cells had attached, the original medium was removed and fresh medium with Xn or vehicle (0.5% v/v DMSO) was added to the plate. After 24 h incubation with 0–25 μM Xn, the medium was removed and fresh medium with cell counting kit-8 solution (Dojindo Laboratories, Shanghai, China) was added. After 1 h incubation, the plate was read by a microplate reader (Tecan M1000, Männedorf, Switzerland) at 450 nm. The cell viability of a well was the absorbance of the well with cell and cell culture medium subtracted from the well with cell culture medium only. The relative cell viability was the viability of the treated cell normalized by the viability of the control (vehicle).

### ELISA of Aβ_1-40_ and Aβ_1-42_

Levels of Aβ_1-40_ or Aβ_1-42_ in medium or cell lysates of N2a/WT and N2a/APP cells were tested with a commercially available sandwich ELISA kit (R&D system, Shanghai, China) following the kit instructions.

### Western-Blot Analysis

After 24 h of drug treatment, cells were washed two times with cold PBS and 200 μL IP lysis buffer added per culture flask, placed on ice for 30 min, scraped from the flasks and collected in 1.5-mL tubes, and centrifuged at 18,000 *g* at 4°C for 20 min. BCA Protein Assay Reagent (Thermo Fisher Scientific) was used to measure total protein concentration of debris-free supernatants. Total protein (20 μg) was boiled for 8 min in 5 × SDS loading buffer (Thermo Fisher Scientific), separated by 10% SDS-polyacrylamide gel electrophoresis, and transferred to polyvinylidene difluoride (PVDF) membranes. Non-specific binding was prevented by incubating membranes in 5% non-fat milk dissolved in 1 × TBST buffer at RT for 1 h. The membranes were incubated overnight at 4°C with solutions of primary antibody diluted with 1 × TBST (see **Table 1**). The membranes were washed and incubated with anti-mouse, anti-rabbit or anti-goat IgG conjugated to horseradish peroxidase (HRP) (1:3000) at RT for 1 h before development in ECL solution (Thermo Fisher Scientific). The densitometry of the blots was quantified by ImageQuant 1D software (GE Healthcare, Pittsburgh, PA, United States).

### Comparative Proteomics

#### Protein Preparation and Labeling

After 24 h of drug treatment, cells were washed twice with cold PBS and dissolved in 600 μL 1 × DIGE lysis buffer (7 M urea, 2 M thiourea, 30 mM Tris-HCl, 4% CHAPS, pH 8.5) per 75 cm^2^ culture flask, placed on ice for 30 min, then scraped from the flasks and collected in 1.5-mL tubes. The cell suspensions were ultrasonicated for 2 min in cycles (4 s on and 6 s off) at 45% power with a Fisher 550 Sonic Dismembrator (Pittsburgh, PA, United States) until the samples were pellucid. Samples were incubated on ice for 30 min and then centrifuged at 20,000 *g* at 4°C for 60 min. The supernatants were ultrafiltered at 14,000 *g* at 4°C for 30 min to remove salt and other impurities, and then diluted in 1 × DIGE lysis buffer. The protein solutions were collected and stored at -80°C. The 2-D Quant Kit (GE Healthcare, United States) was used to measure protein concentrations according to the manufacturer’s protocol.

All the samples from N2a/WT cells and N2a/APP cells treated with or without Xn were diluted to 5 μg/μL after protein quantification. The protein samples (25 μg) were labeled with Cy3 or Cy5, and the internal standards derived from the mixture of all samples were labeled with Cy2. Each vial of CyDye, Cy2 (GE Healthcare, 25-8008-62), Cy3 (GE Healthcare, 25-8008-61), and Cy5 (GE Healthcare, 25-8008-62), was dissolved in 99.8% anhydrous *N, N*-dimethylformamide (DMF) (Sigma 227056) to obtain a stock concentration of 1 nmol/μL. DMF was added to obtain working solutions of 200 pmol/μL of each CyDye with which to label protein samples. Labeling reactions were carried out on ice in the dark and were quenched by adding 1 μL 10 mM lysine at 4°C in the dark for 10 min. After protein labeling, the Cy2-, Cy3-, Cy5-labeled samples were mixed together and rehydration buffer [7 M urea, 2 M thiourea, 4% CHAPS, 2% DTT, 2% (v/v) IPG buffer, pH 3-11 NL] added to a total sample volume of 450 μL.

#### Protein Separation

The first-dimension protein separation employed the Ettan IPGphor Isoelectric Focusing (IEF) System (GE Healthcare). For each separation, 75 μg protein were transferred on 24-cm pH 3-11 NL ImmobilineDryStrips (GE Healthcare). Then, 1.5 mL mineral oil was added to cover each strip to reduce solvent evaporation. Proteins were immobilized onto strips at 50 V for 18 h, followed by focusing at 300 V for 12 h, 500 V step for 2 h, 1000 V gradient for 2 h, 8000 V gradient for 8 h, 8000 V step for 8 h at 20°C. The temperature for IEF was kept at 18°C. After IEF, each strip was immediately equilibrated in a 15 ml buffer of 6 M urea, 75 mM Tris-HCl buffer (pH 8.8), 30% (v/v) glycerol, 2% (w/v) SDS, and 1% (w/v) DTT for 15 min at RT on a shaking table, and subsequently re-equilibrated in the same buffer containing 6 M urea, 75 mM Tris-HCl buffer (pH 8.8), 30% (v/v) glycerol, 2% (w/v) SDS, and 4.5% (w/v) IAA for 15 min. The equilibrated strips were loaded on the top of 12.5% SDS-PAGE gels and covered with 0.5% (w/v) ultralow-melting-point agarose sealing solution [25 mM Tris, 192 mM glycine, 0.1% SDS, 0.5% (w/v) agarose, 0.002% (w/v) bromophenol blue]. Electrophoresis was executed with an Ettan DALTsix Electrophoresis System (GE Healthcare) at 1 W/gel for 1 h, 11 W/gel for 4.5 h at 12.5°C in the dark. After peptide separation in the second dimension had been completed, the gels were immediately scanned with a Typhoon TRIO Variable Mode Imager (GE Healthcare).

### Image Analysis

The DIGE gels were analyzed with the DeCyder software package (Version 6.5 GE Healthcare). After confirmation of appropriate spot detection, matching, and normalization, the spot statistics were reviewed. Both DeCyder and Progenesis employed one-way ANOVA to quantify differential expression of spots among the experimental groups. The normalized spot density was further compared across the gels of the replicate groups. Protein spots found to be statistically significant (*p* ≤ 0.05) were isolated for further analysis.

### Protein Identification

Protein (1.4 mg) was separated by the same DIGE method but without protein labeling. The gel was stained with Coomassie blue solution (0.12% Coomassie Brilliant Blue G-250, 20% ethanol, 10% phosphoric acid, 10% ammonia sulfate). Proteins displaying significant variation (*P* ≤ 0.05) were manually excised from the blue-stained gel and quenched with 50% acetonitrile in 25 mM ammonium bicarbonate followed by dehydration in 100% acetonitrile. After the reagents had been removed, the gel pieces were digested with 0.15 μg of sequencing-grade trypsin (Promega, Madison, WI, United States) in 15 μL digestion buffer containing 25 mM ammonium bicarbonate. The mixture was incubated overnight at 37°C and then subjected to analysis using mass spectrometry (MS).

Peptide analysis was performed by MALDI-TOF-MS/MS (SCIEX TOF/TOF 5800 System, ABSCIEX, Framingham, MA, United States) analysis was used for peptide analysis. In brief, 1 μL of peptide extraction was crystallized with 0.7 μL 10 mg/mL α-cyano-4-hydroxycinnamic acid (CHCA) in 0.1% trifluoroacetic acid (TFA), 50% acetonitrile (ACN) on the target and dried at room temperature. The spectra were externally calibrated and MASCOT (Matrix Science, United Kingdom) was adapted to search the Swiss-Prot database for mouse brain proteins. The search was conducted with a tolerance on mass measurement of 100 ppm in MS mode and 0.3 Da in MS/MS mode. Protein molecular weight (MW) was also used for protein identification based on the location of the excised protein spot from the 2-D gel.

### Immunocytochemistry

N2a/WT and N2a/APP cells were seeded on coverslips and treated with Xn or vehicle for 24 h. The cells on the coverslip were fixed for 10 min in cold 4% paraformaldehyde (PFA), rinsed three times with 1× PBS and then permeabilized in 0.3% Triton X-100 in 1× PBS for 30 min. After fixation, the cells were sealed in 5% BSA diluted in 1× PBS for 30 min. Cells were incubated with primary antibodies 8-OHdG overnight at 4°C, then with peroxidase-labeled donkey-anti-goat secondary antibody for 1 h in the dark at RT, then rinsed with 1× PBS four times. Next, DAPI (4′, 6-diamidino-2-phenylindole) was added to the cells for 5 min in the dark and then rinsed four times with 1× PBS. Finally, Fluo-Antifading Medium (Beyotime, Beijing, China) was added to the microslide and a coverslip added. The cells were examined by laser confocal microscopy.

### Bioinformatics Analysis and Statistics

Functional annotation of differentially expressed proteins was performed with the Database for Annotation, Visualization and Integrated Discovery Resource (DAVID^1^). Gene ontology (GO) terms for biological processes (BP), molecular functions (MF), charts and cellular components (CC), were obtained with default statistical parameters.

Results are expressed as means ± SEM. One-way ANOVA was used to determine the statistical significance of differences among the groups and following *post hoc* assessment by the Student–Newman–Keuls Method (GraphPad Prism 7.0^2^) except special indication. A *p*-value less than 0.05 was considered to be statistically significant. Detailed *p*-value of analyses are listed in the **Supplementary Tables S6–S9**.

## Results

### Xn Has Low Cytotoxicity for N2a/WT and N2a/APP Cells

We first studied the cytotoxicity of Xn on both N2a/WT and N2a/APP cells. Compared with vehicle control (0.5% DMSO), no difference in cell viability was observed in N2a/WT cells treated for 24 h with less than or equal to 12.5 μM Xn (**Figure 1B**). For N2a/APP cells, loss of cell viability was seen in concentrations exceeding 5.0 μM Xn treatment (**Figure 1C**). Accordingly, treatment for 24 h with a maximum concentration of 3.0 μM Xn was used in the following investigations.

### Xn Inhibited Aβ Accumulation and APP Processing

**FIGURE 2:**
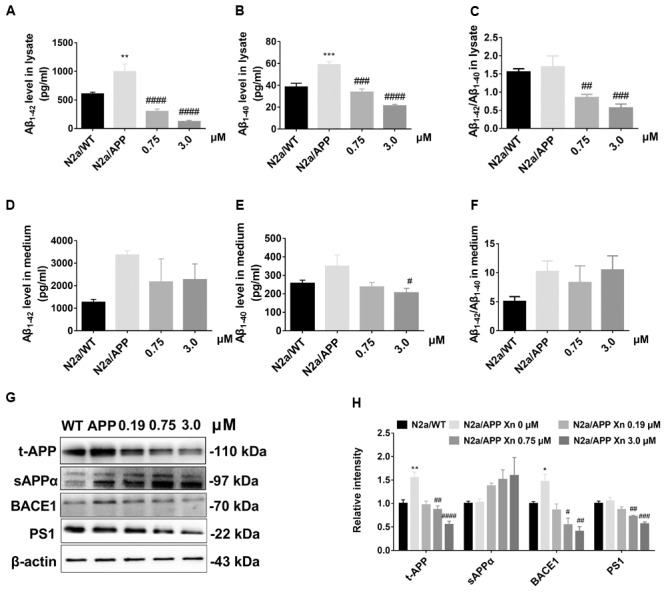
Xn inhibited Aβ accumulation and APP processing. Levels of Aβ_1-42_ **(A,D)**, Aβ_1-40_ **(B,E)**, and Aβ_1-42_/Aβ_1-40_ **(C,F)** of cell lysate **(A–C)** and cell culture media **(D–F)** as a function of Xn concentration were determined by ELISA. Ratios of Aβ_1-42_/Aβ_1-40_ are shown on the right **(C,F)**. Levels of t-APP, s-APPα, BACE1, and PS1 were determined by Western-blot analysis **(G,H)**. β-Actin was used as a loading control. *N* = 5 for **(A–C)**; *N* = 4 for t-APP and BACE1 **(G)**; *N* = 3 for the rest. ^∗^*p* < 0.05, ^∗∗^*p* < 0.01 compared with N2a/WT cells. ^#^*p* < 0.05, ^##^*p* < 0.01, ^###^*p* < 0.001, ^####^*p* < 0.0001 compared with untreated N2a/APP cells.

To study the effect of Xn on Aβ accumulation, we determined the levels of Aβ_1-42_ and Aβ_1-40_ in lysates and culture medium of N2a/WT cells and N2a/APP cells with or without 24 h Xn treatment. Shown in **Figures 2A,B**, the level of “toxic” Aβ_1-42_ and “non-toxic” Aβ_1-40_ in N2a/APP cell lysates were both one third higher than that in N2a/WT cell lysates. The ratio of Aβ_1-42_ to Aβ_1-40_ in N2a/APP cell lysates showed a trend of modest increase compared to that in N2a/WT cell lysates (**Figure 2C**). The levels of both Aβ_1-42_ and Aβ_1-40_ in N2a/APP cell culture medium were higher than that in N2a/WT cell culture medium yet the differences are not as big as those in cell lysates (**Figures 2D,E**). After 0.75 and 3 μM Xn treatment for 24 h, N2a/APP cell lysates had significant reduction in Aβ_1-42_, Aβ_1-40_, and Aβ_1-42_ to Aβ_1-40_ ratio compared with those from control treated cells (**Figures 2A–C**). Yet the reductions of Aβ_1-42_, Aβ_1-40_, and Aβ_1-42_ to Aβ_1-40_ in cell medium were not significant (**Figures 2D–F**) except the level of Aβ_1-40_ in 3 μM Xn-treated cells (**Figure 2F**). The data from ELISA analyses show the potency of Xn in reducing Aβ generation.

Since Xn treatment suppressed Aβ generation by N2a/APP cells, we used Western-blot analysis (**Figures 2G,H**) to explore the critical proteins implicated in APP processing. Shown in **Figures 2G,H**, N2a/APP cells had significantly higher mean levels of total APP and BACE1 than N2a/WT cells. N2a/APP cells treated with 3.0 μM Xn had significantly reduced levels of APP, BACE1, and PS1 compared with vehicle-treated N2a/APP cells. The mean level of sAPPα in Xn-treated vs. vehicle-treated N2a/APP cells increased with Xn concentration (**Figures 2G,H**). The data from Western-blot analyses suggested the effect of Xn in a non-amyloidogenic pathway in N2a/APP cells.

### Xn Attenuated Tau Phosphorylation in Both N2a/APP Cells and HEK293/Tau Cells

**FIGURE 3:**
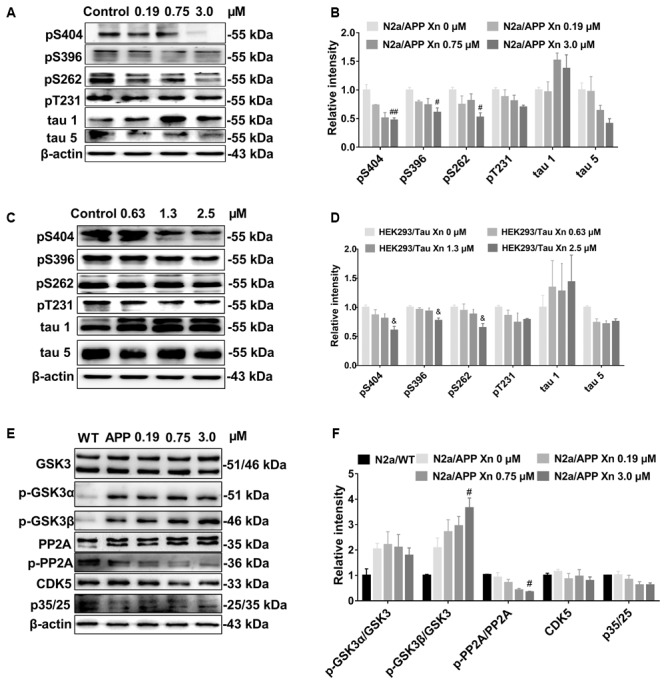
Xn attenuated tau phosphorylation in both N2a/APP cells and HEK293/Tau cells. Shown are the levels of phosphorylated tau and total tau in N2a/APP cells **(A,B)**, the levels of phosphorylated tau and total tau in HEK293/Tau cells; **(C,D)**. The phosphorylation of upstream pathways of tau in N2a/WT and N2a/APP cells **(E,F)** were determined by Western-blot analysis. β-Actin was used as a loading control. *N* = 3. ^#^*p* < 0.05 and ^##^*p* < 0.01 compared to untreated N2a/APP cells, ^&^*p* < 0.05 compared to untreated HEK293/tau cells.

To investigate the effect of Xn on tau phosphorylation, we profiled various sites in phosphorylated tau using Western-blot analysis. Shown in **Figures 3A,B**, N2a/APP cells treated with 3.0 μM Xn showed significant reduction of phosphorylated tau at serine 404, 396, and 262. The effect of Xn on phosphorylated tau at site threonine 231 in N2a/APP cells was not significant as other sites above.

We validated the effect of Xn treatment in another cell line with tau hyper-phosphorylation, namely the HEK293/Tau cell line. Shown in **Figures 3C,D** are responses of HEK293/Tau to Xn similar to those observed with N2a/APP cells. HEK293/Tau cells treated with 3.0 μM Xn had significant reductions in phosphorylated tau at serine 404, 396, and 262.

While many kinases and phosphatases are responsible for the phosphorylate state of tau, we focused on protein pathways most often associated with tau phosphorylation, namely p-GSK3β, PP2A, p35/25, and CDK5 (**Figures 3E,F**). N2a/APP cells treated with 3.0 μM Xn had significantly higher levels of p-GSK3β (Ser 9) and significantly lower levels of p-PP2A while having no significant effects on the levels of p35/25, co-activators of CDK5.

### Xn Modified Critical Proteins Involved in ER Stress and Oxidative Stress

**FIGURE 4:**
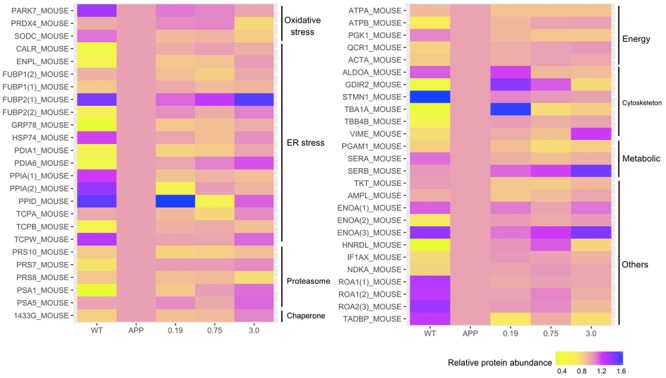
Xn treatment altered the presence of 51 proteins including: endoplasmic reticulum (ER) stress-associated proteins; oxidative stress-associated proteins; proteasome-associated proteins; ATPase and metabolism-associated proteins; cytoskeleton-associated proteins; molecular chaperones-associated proteins, and others.

**FIGURE 5:**
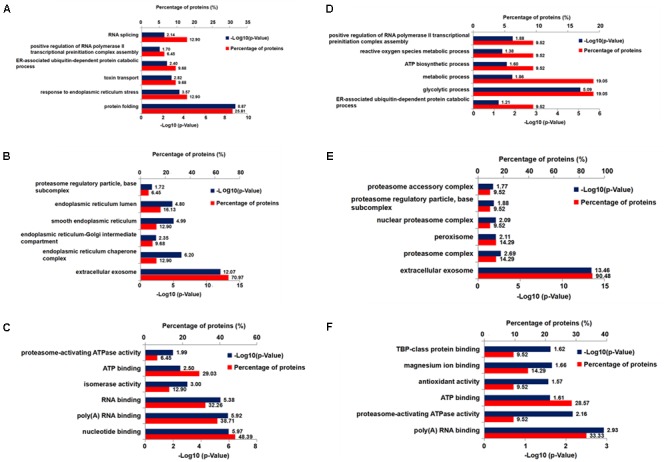
Six bioinformatics analyses of differentially expressed proteins from Xn-treated and-untreated N2a/APP cells. Shown are **(A)** the biological process, **(C)** the molecular function, and **(B)** the cellular component enrichment in Gene Ontology terms of the differentially expressed proteins in N2a/APP cells (when compared with N2a/WT cells); **(D)** the biological process, **(F)** the molecular function, and **(E)** the cellular component enrichment of the differentially expressed proteins in Xn-treated N2a/APP cells (when compared with untreated N2a/APP cells).

To explore molecular species affected by Xn treatment, we performed a comparative proteomic analysis using 2D-DIGE peptide separation and identification by MS. A total of 51 proteins (shown in **Figure 4**) in 2D-DIGE gels was significantly (adjust *p*-value < 0.05 in one way ANOVA) different in any four comparison pairs (N2a/APP vs. N2a/WT, or 0.19 μM Xn vs. N2a/APP, or 0.75 μM Xn vs. N2a/APP, or 3.0 μM Xn vs. N2a/APP). Thirty of the 51 differentially expressed lysate proteins distinguished N2a/APP from wild-type (WT) N2a cells (N2a/WT), and an additional 21 lysate proteins characterized differences in N2a/APP cells in the presence and absence of Xn. The 51 proteins were grouped in seven categories: ER stress-associated proteins; oxidative stress-associated proteins; proteasome pathway-related proteins; cytoskeleton-associated proteins; molecular chaperones; energy metabolism and others. We performed functional annotations using DAVID to gain further insight into the 51 differentially expressed proteins. The major categories of the differentially expressed proteins in N2a/APP cells were; “protein folding,” “response to ER stress,” and “toxin transport” in biological process (**Figure 5A**); “extracellular exosome,” “ER chaperone complex,” and “ER-Golgi intermediate compartment,” and “nucleotide binding” in the CC (**Figure 5B**), and “poly(A) RNA biding,” and “RNA binding” in MF (**Figure 5C**). The major categories of differentially expressed proteins in Xn-treated N2a/APP cells were: “ER associated ubiquitin-dependent protein catabolic process,” “glycolytic process,” and “metabolic process” in biological process (**Figure 5D**); “extracellular exosome,” “proteasome complex,” and “peroxisome” in the CC (**Figure 5E**), and “poly (A) RNA binding,” “proteasome-activating ATPase activity,” and “ATP binding” in MF (**Figure 5F**). The finding from the functional enrichment supported our finding that Xn modified proteins relevant to ER stress, oxidative stress and metabolic dysfunction.

We also identified some coincident proteins in the four comparison pairs (shown in **Supplementary Figure S1** and **Supplementary Tables S1–S5**), including ER stress-related proteins, protein disulfide-isomerase (PDA1), proteasome-related proteins, 26S protease regulatory subunit 10B (PRS10) and 26S protease regulatory subunit 8 (PRS8), energy metabolism-related protein, ATP synthase subunit alpha (ATPA), and transketolase (TKT) and alpha-enolase (ENOA).

**FIGURE 6:**
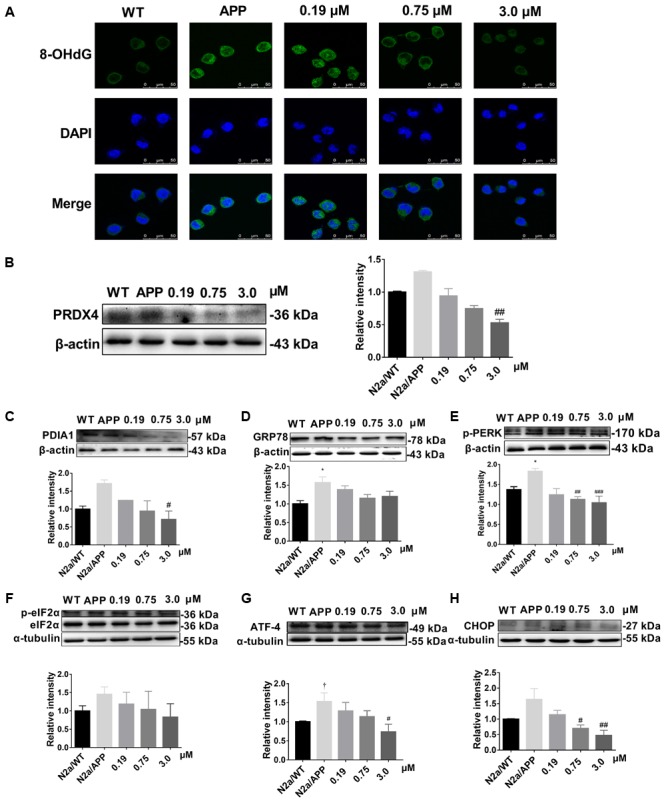
Xn reduced oxidative stress and ER stress of N2a/APP cells. **(A)** 8-OHdG (in green) immune staining of N2a/WT and N2a/APP cells. DAPI (in blue) stained the cell nuclei. Proteins PRDX4 **(B)**, PDIA1 **(C)**, GRP78 **(D)**, p-PERK **(E)**, p-ELF2α **(F)**, ATF-4 **(G)**, and CHOP **(H)** in N2a/WT and N2a/APP cells were determined by Western-blot analyses. β-Actin was used as a loading control for PRDX4, GRP78, PDIA1, and p-PERK. α-Tubulin was used as a loading control for ATF-4 and CHOP. Total ELF2α was used as a loading control for p-ELF2α. *N* = 4 for p-PERK, 3 for the rest. ^∗^*p* < 0.05 compared with N2a/WT cells, ^#^*p* < 0.05, ^##^*p* < 0.01, ^###^*p* < 0.001 compared to untreated N2a/APP, ^†^*p* = 0.06 compared with N2a/WT cells in Student *T*-test.

As supplemental and validating data, the expression levels of: peroxiredoxin-4 (PRDX4) (**Figure 6B** and **Supplementary Figure S4**), protein disulfide isomerase (PDIA1) (**Figure 6C** and **Supplementary Figure S3**), the ER chaperone protein GRP78, (**Figure 6D** and **Supplementary Figure S2**), and the phosphorylated-pancreatic ER eIF2α kinase (p-eIF2α) (**Figure 6F**) were determined by Western-blot analysis. Expression levels of PRDX4, PDIA1, GRP78 were higher in N2a/APP cells compared with N2a/WT cells and lower in Xn-treated N2a/APP cells compared with vehicle-treated N2a/APP cells. These three proteins showed consistent trends on analyses using Western-blot and proteomics methodologies.

### Xn Reduced Oxidative Stress and ER Stress of N2a/APP Cells

Given our findings by comparative proteomics that Xn treatment impacted critical proteins involved in oxidative stress and ER stress, we asked whether Xn could modulate indicators of oxidative stress (8-hydroxy-2′-deoxyguanosine, 8-OHdG) and ER stress [unfolded protein response (UPR)]. Shown in **Figure 6A**, 8-OHdG had higher expression in N2a/APP cells (second column) compared with N2a/WT cells (first column). N2a/APP cells treated with 0.75 μM (third column) and 3.0 μM Xn (fourth column) showed lower levels of 8-OHdG compared with N2a/APP cells treated with vehicle, implying that Xn reduced oxidative stress in N2a/APP cells. To study whether Xn impacted the UPR in N2a/APP cells, we profiled critical proteins in the UPR pathway, namely activating transcription factor-4 (ATF-4), transcriptional factor C/EBP homologous protein (CHOP) and phosphorylated eukaryotic initiation factor-2 alpha (p-eIF2-α). N2a/APP cells had modestly higher levels of phosphorylated PERK (**Figure 6E**), eukaryotic translation initiation factor 2 subunits (eIF2-α) (**Figure 6F**), ATF-4 (**Figure 6G**), and CHOP (**Figure 6H**). By contrast, N2a/APP cells treated with Xn showed concentration-dependent stepwise lower levels of p-eIF2-α (**Figure 6F**) and significantly reduced levels of ATF-4 (**Figure 6G**) and CHOP (**Figure 6H**). These results, together with the protein levels of PDIA1 (**Figure 6C**) and GRP 78 (**Figure 6D**), suggested that Xn treatment attenuated ER stress in N2a/APP cells.

## Discussion

We examined the action of xanthohumol (Xn) on murine neuroblastoma N2a cells stably expressing human Swedish mutant APP, a well-characterized cellular model of AD. We found higher levels of Aβ and the amyloidogenic pathway, which was consistent with a previous report (Yan et al., 2012). Xn reduced the cellular burden of Aβ_1-42_, which is cytotoxic and has highest propensity for aggregation, Aβ_1-40_ which is less cytotoxic and less propensity for aggregation (Meisl et al., 2014), and the ratio of Aβ_1-42_ to Aβ_1-40_ which is widely used in clinical diagnosis (Janelidze et al., 2016). Further, Xn suppressed the levels of total APP, BACE1 that are responsible for N-terminal cleavage for production of Aβ (Vassar et al., 1999) and PS1 that is responsible for C-terminal cleavage for production of Aβ (De Strooper et al., 1998). Besides, Xn suppressed the hyperphosphorylation of tau at the sites Ser 404, Ser 396, and Ser 262, which are critical for formation of paired helical filaments (Goedert, 1996). The fact that Xn robustly increased phosphorylated GSK-3β is consistent with a previous report (Lv et al., 2017) describing Xn activation of GSK-3β via the AMPK pathway. Xn treatment attenuated the two major characteristics of AD, namely, Aβ levels and tau hyperphosphorylation. Furthermore, proteomics and functional analyses revealed that Xn modulated 51 proteins that involve oxidative stress, ER stress, proteasomal function, chaperone, cytoskeleton, ATPase and Metabolism. Detailed discussion is as follows.

### Oxidative Stress-Related Proteins and Oxidative DNA Damage

Our results suggest that Xn relieves oxidative stress, which is consistent with previous reports (Dorn et al., 2013; Lv et al., 2017). The category “oxidative stress-related proteins” includes protein DJ-1 (PARK7), PRDX4 and superoxide dismutase [Cu-Zn] (SODC), two (PRDX4, SODC) of which were reduced in N2a/APP cells treated with the higher concentrations of Xn. Additionally, the intensity of PRDX4 (a peroxiredoxin that removes peroxides) was increased in N2a/APP vs. N2a/WT cells, which is consistent with oxidative stress, and was reduced with Xn treatment. Further evidence of oxidative stress in N2a/APP vs. N2a/WT cells included the higher levels of 8-OHdG in the AD-transgenic line and Xn-associated reductions in this DNA damage marker. 8-OHdG adducts are generated when ROSs, the most common of which is the hydroxyl radical, react with nuclear and mitochondrial DNA, such that urine 8-OHdG is a reliable index of DNA oxidation and a potential biomarker of early cellular dysfunction in AD (Valavanidis et al., 2009; Zhang and Rissman, 2017).

### ER Stress-Related Proteins and Proteasomal Proteins

Accumulation of Aβ and tau is associated with perturbations in the UPR in AD and a number of other progressive neurodegenerative diseases (Stutzbach et al., 2013). We found perturbations of 18 UPR-related proteins in N2a/APP vs. N2a/WT cells. Some of these proteins are critical in proteolysis and AD progression. We found levels of three 26S protease regulatory subunits (PRS7, PRS8, and PRS10) that were higher in N2a/APP cells and lower in Xn-treated cells. An ATPase (MS37) that acts as a regulatory subunit of the 26S proteasome is reported to immune react with NFTs, plaque neurites and neuropil threads in the hippocampus of AD brains (Fergusson et al., 1996). Another example is peptidyl-prolyl *cis*–*trans* isomerase, which has been shown to be decreased in expression (Sultana et al., 2007) and inactivated (Lee et al., 2011) in AD brains. We also found lower expression of two subunits of peptidyl-prolyl *cis*–*trans* isomerase (PPID and PPIA) in N2a/APP cells, and the level of PPID was restored in Xn-treated cells.

Our proteomics study revealed some intriguing expressions of proteins in the categories of ER stress-related proteins and proteasomal proteins. For example, calreticulin (CALR) has been reported to have low immune reactivity and less CALR mRNA in AD brains (Taguchi et al., 2000); however, expressed levels of this protein in N2a/APP cells were higher than in N2a/WT cells. As another study revealed (Bollo et al., 2010), CALR could be crucial to cell-protective mechanisms against ER stress, as the level of CALR rose rapidly when cells were treated with increasing levels of an ER stressor. Thus, the high expression of CALR in N2a/APP cells suggests the presence of elevated ER stress. Indeed, our proteomics and Western-blot analyses (**Figure 6**) show clear evidence of ER stress in N2a/APP cells and relief of ER stress in these cells by Xn treatment.

The unfolded protein response is reported to have an important role in the prevention and attenuation of AD and Parkinson disease progression by preventing neuronal accumulation of misfolded proteins (Milisav et al., 2015). In the UPR cascade, ATF-4 and PERK, which are activated by dissociation of GRP78/BIP, serve as two of the three classes of main ER stress sensors. Phosphorylated PERK initiates phosphorylation of ElF2α, and PDIA1 assists by reducing proteins that have improper paired disulfide bonds. Thus, in response to ER stress, levels of ATF-4, p-PERK, p-elF2α, and PDIA1 would be expected to be increased, as we observed in N2a/APP vs. N2a/WT cells. Importantly, Xn treatment was associated with reduced levels of the aforementioned proteins. Taken together, therefore, our finding that Xn suppressed ER stress is consistent with a previous study showing the modulation of ER stress in the step of Xn activation of the AMPK pathway (Zimmermann et al., 2015).

### Cytoskeletal Proteins

The category of cytoskeletal proteins contains some proteins that closely interact with Aβ and tau. For example, the intermediate filament protein vimentin, which co-localizes with Aβ in the cerebral cortex, cerebellum and hippocampus of AD brains (Levin et al., 2009), was increased in N2a/APP vs. N2a/WT cells and suppressed in N2a/APP cells treated with Xn (0.19 and 0.75 μM). Stathmin (STMN1), a microtubule-destabilizing neuroprotein, was more heavily expressed in N2a/WT cells than in N2a/APP cells, consistent with loss of support for potential neuritic processes in the AD culture model. Similarly, in the frontal and temporal cortex of AD brain, STMN1 has a reduced expression and preferentially localizes in NFTs (Jin et al., 1996; Cheon et al., 2001), Importantly, the level of STMN1 in N2a/WT cells was higher with Xn treatment (0.19 and 0.75 μM), which suggests the chalcone may have been acting to maintain the neuronal cytoskeleton which, *in vivo*, may serve to preserve synaptic plasticity (Uchida et al., 2014).

Taken together, Xn-associated modification of expression of these cytoskeletal proteins may correlate with the suppression of Aβ accumulation and tau hyperphosphorylation upon treatment of N2a/APP cells with Xn.

## Conclusion

**FIGURE 7:**
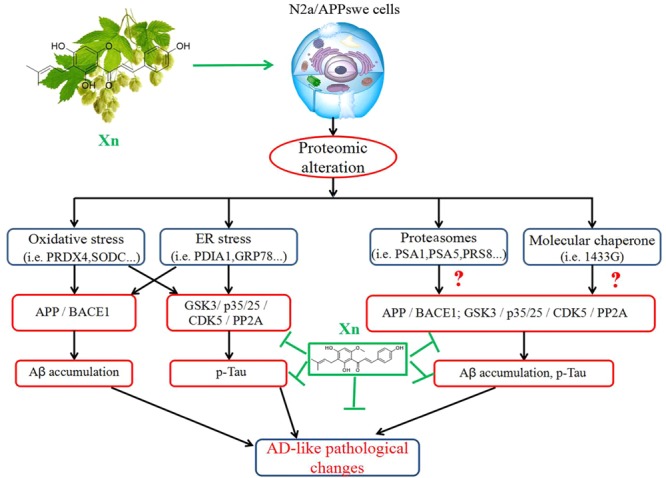
The mode of action of xanthohumol. Xn treatment suppressed AD-related changes, notably Aβ and tau phosphorylation via APP processing and the GSK-3β and PP2A pathways, which may be attributed to modifications of proteins related to functions of oxidative stress, ER stress, proteasomes and molecular chaperones.

Xanthohumol significantly suppresses Aβ production and tau hyperphosphorylation in N2a/APP cells via APP processing and the GSK-3β pathway, as envisaged in the cascade of molecular events shown schematically in **Figure 7**. Comparative proteomics and functional studies revealed that Xn induced modulation of redox regulation proteins, ER, proteasomal, and cytoskeleton/cytoplasmic proteins, which may correlate with the suppression of Aβ production and tau hyper-phosphorylation in this cell model of AD. Xn treatment also altered the expression of other proteins, such as those involved in energy regulation and metabolism.

Taken in concert, these *in vitro* findings suggest that Xn is a promising candidate for AD therapy that merits prompt evaluation for efficacy and safety in animal models of AD. The ability of Xn to modulate multiple proteins, pathways and functions to effect reduction of AD-like pathology expressed by N2a/APP cells might have advantages in treating this complex neurodegenerative disease. This view is consistent with the therapeutic failures of drug candidates that target specific AD-related molecules, such as immune therapies or inhibitors directed at β- or γ-secretases (Kikuchi et al., 2017; Moussa, 2017).

While Xn appears to have multiple physiological and therapeutic potential, any effective secondary prevention or treatment of AD will require long-term therapy with minimal adverse side effects. In this regard, it is noteworthy that Xn, acting as a weak electrophile Michael acceptor, is thought to exert chemopreventive effects via induction of detoxification enzymes such as NADPH quinone oxidoreductase 1 (NQO1) (Dietz and Bolton, 2011), which has also been associated with AD (Chhetri et al., 2017).

## Author Contributions

XaH, JW, XC, and PL performed the experiments, analyzed the data, and drafted the manuscript. SW, FS, ZZ, FZ, XnH, JL, and XY designed the study and analyzed the data. GS, PS, and XY wrote and revised the manuscript. The authors would like to thank Mr. Joshua Bojie Liu for the technical support in bio-informatics analysis.

## Conflict of Interest Statement

The authors declare that the research was conducted in the absence of any commercial or financial relationships that could be construed as a potential conflict of interest.

## Footnotes

1 https://david.ncifcrf.gov

2 http://www.graphpad.com/

## Supplementary Material

FIGURE S1 No protein **(a)** was changed in four comparison groups. One protein (ATPA) **(b)** was changed in various concentrations of Xn-treated N2a/APP cells compared with untreated N2a/APP cells.

FIGURE S2 The MALDI-TOF-MS map of GRP78. **(a)** MS/MS spectrum fingerprint of GRP78; **(b)** m/z 1512.792; **(c)** m/z 2149.051.

FIGURE S3 The MALDI-TOF-MS map of PDIA1. **(a)** MS/MS spectrum fingerprint of PDIA1; **(b)** m/z 1833.826; **(c)** m/z 2092.982.

FIGURE S4 The MALDI-TOF-MS map of PRDX4. **(a)** MS/MS spectrum fingerprint of PRDX4; **(b)** m/z 1624.746; **(c)** m/z 2428.034.

TABLE S1 The coincident proteins between N2a/APP vs. N2a/WT and 0.19 μM/Xn vs. N2a/APP.

TABLE S2 The coincident proteins between N2a/APP vs. N2a/WT and 0.75 μM/Xn vs. N2a/APP.

TABLE S3 The coincident proteins between 3.0 μM/Xn vs. N2a/APP and 0.19 μM/Xn vs. N2a/APP.

TABLE S4 The coincident proteins between 0.75 μM/Xn vs. N2a/APP and 0.19 μM/Xn vs. N2a/APP.

TABLE S5 The coincident proteins between 0.19 μM/Xn vs. N2a/APP and 0.75 μM/Xn vs. N2a/APP and 3.0 μM/Xn vs. N2a/APP.

TABLE S6 P-Value in **Figure 1**.

TABLE S7 P-Value in **Figure 2**.

TABLE S8 P-Value in **Figure 3**.

TABLE S9 P-Value in **Figure 6**.
