# Supplementary figures and images for: The Prenylflavonoid Xanthohumol Reduces Alzheimer-Like Changes and Modulates Multiple Pathogenic Molecular Pathways in the Neuro2a/APPswe Cell Model of AD
Source: Front Pharmacol. 2018 Apr 4;9:199. doi: 10.3389/fphar.2018.00199 (PMC5893754; doi:10.3389/fphar.2018.00199)

a

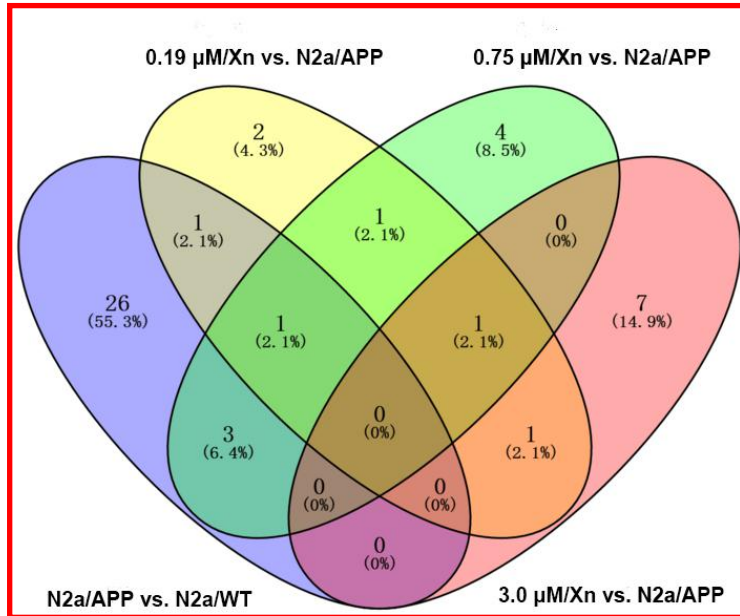

b

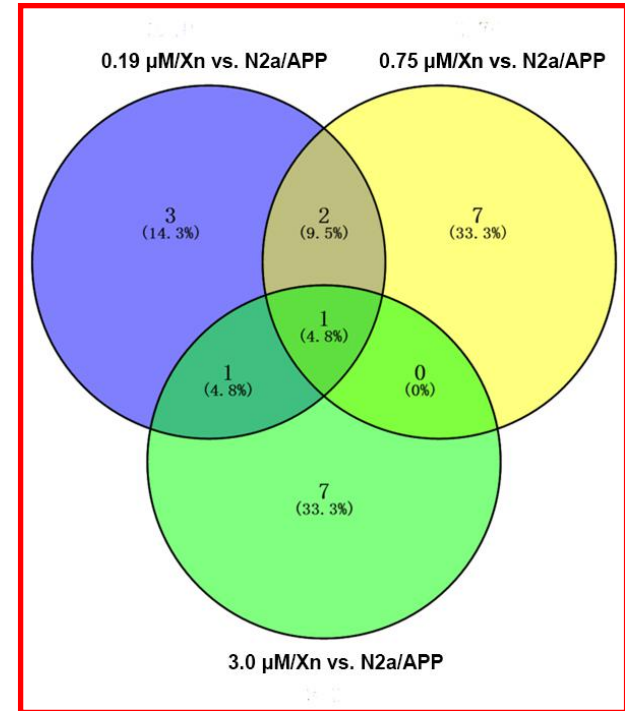

Figure S1

Huang et al., 2017

a

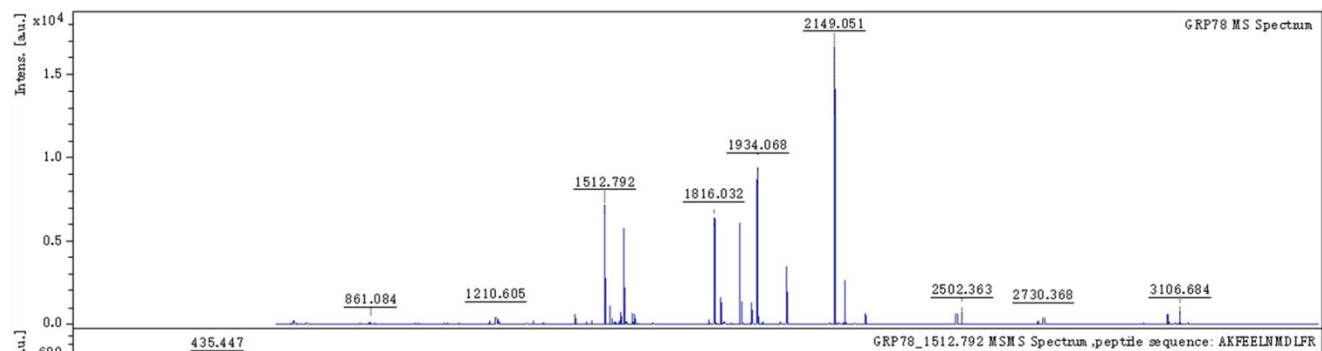

b

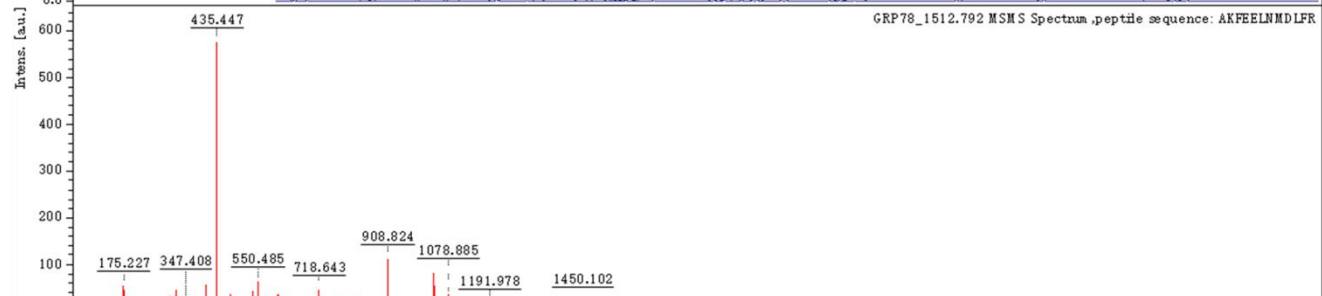

c

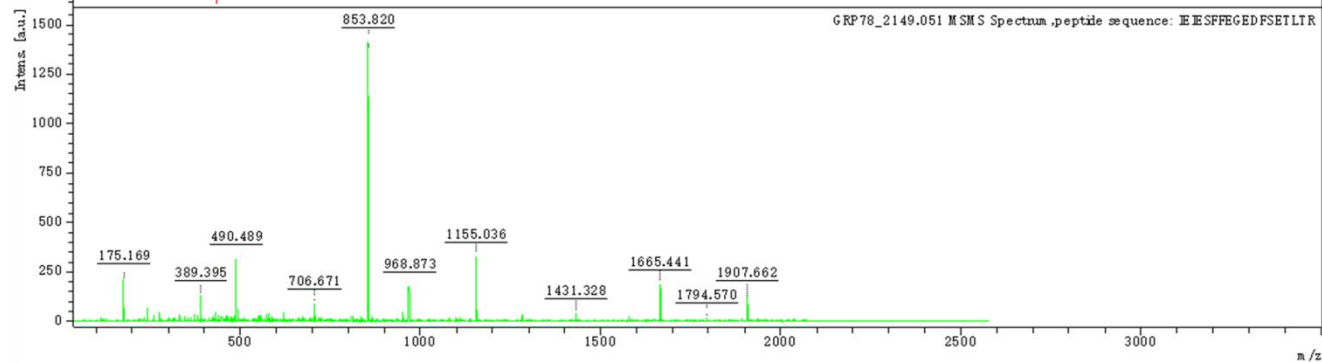

Figure S2

Huang et al., 2017

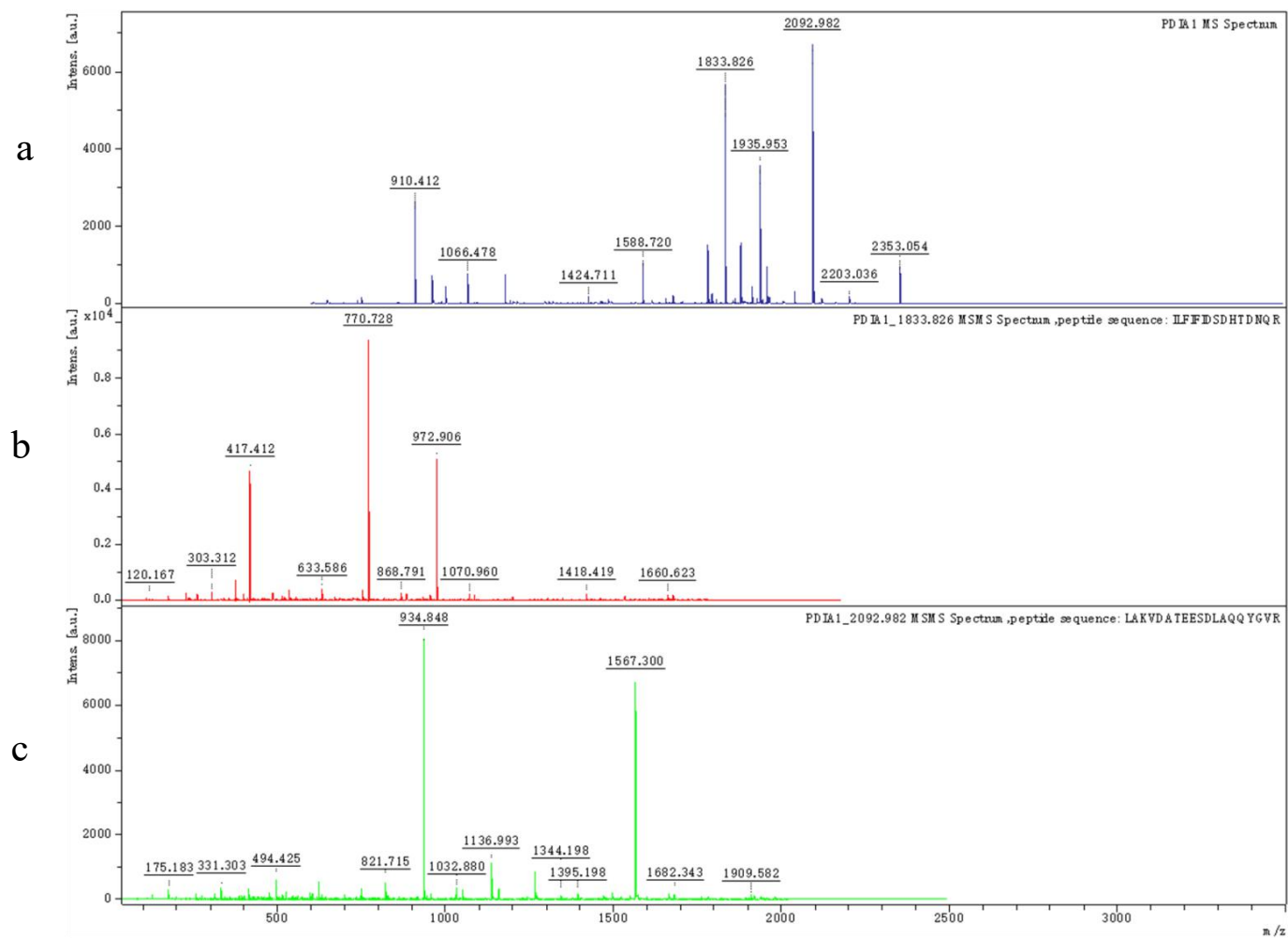

Figure S3

Huang et al., 2017

a

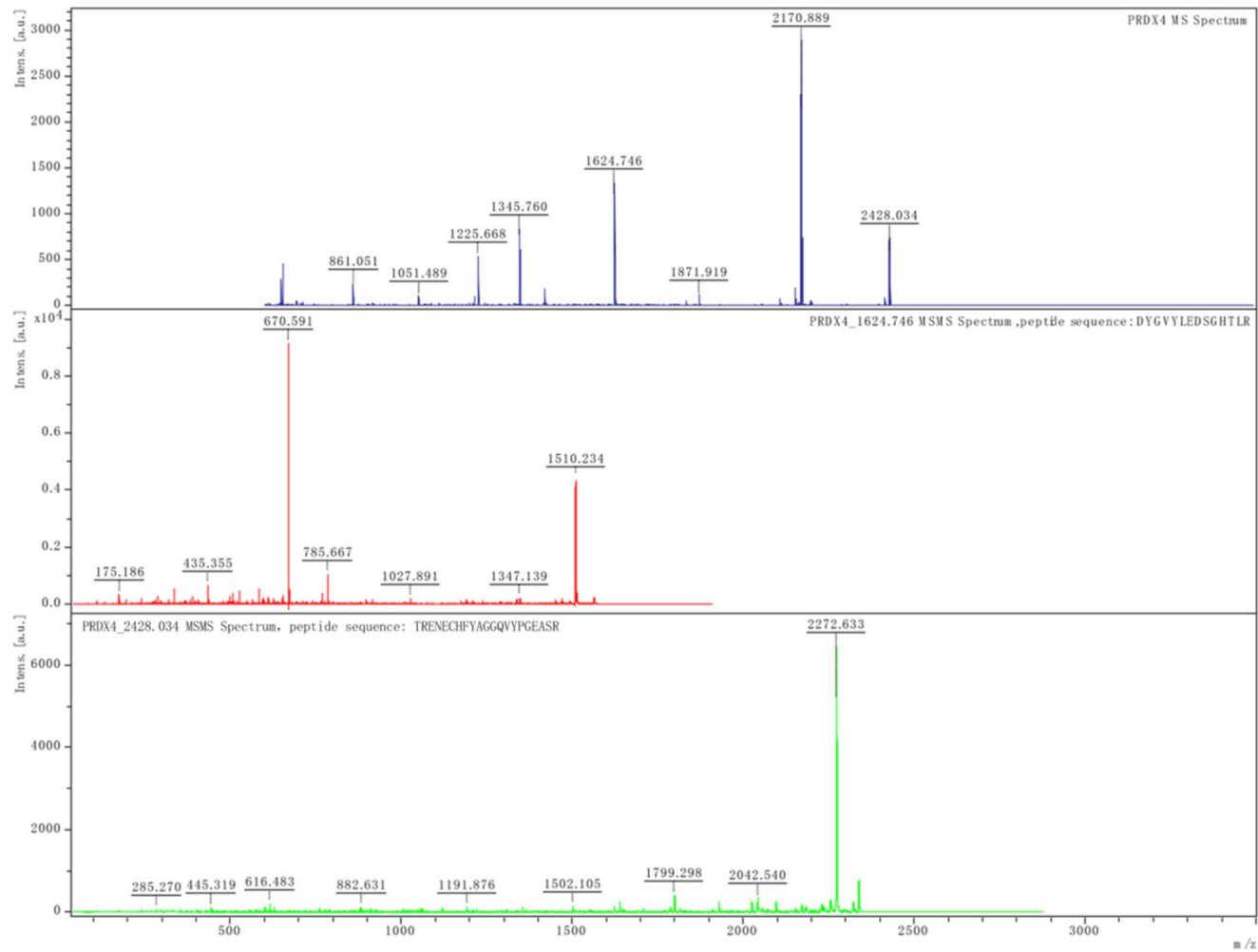

c

Figure S4

Huang et al., 2017

Supplement: FIGURE S1 — No protein (a) was changed in four comparison groups. One protein (ATPA) (b) was changed in various concentrations of Xn-treated N2a/APP cells compared with untreated N2a/APP cells. [file Image_1.PDF]
