# Supplementary material for: The Prenylflavonoid Xanthohumol Reduces Alzheimer-Like Changes and Modulates Multiple Pathogenic Molecular Pathways in the Neuro2a/APPswe Cell Model of AD
Source: Front Pharmacol. 2018 Apr 4;9:199. doi: 10.3389/fphar.2018.00199 (PMC5893754; doi:10.3389/fphar.2018.00199)
Supplement: TABLE S1 — The coincident proteins between N2a/APP vs. N2a/WT and 0.19 μM/Xn vs. N2a/APP. [file Table_1.DOC]

Table S1 Huang et al., 2017

Table S2 Huang et al., 2017

Table S3 Huang et al., 2017

Table S4 Huang et al., 2017


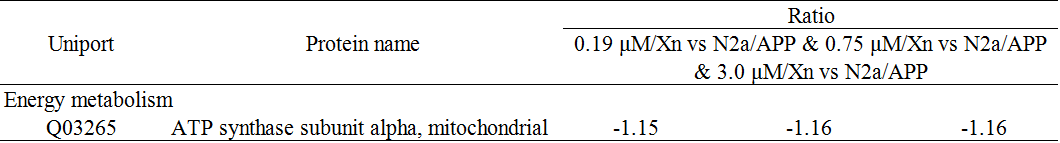


Table S5 Huang et al., 2017

Table S6 Huang et al., 2017

Table S7 Huang et al., 2017

Table S8 Huang et al., 2017

Table S9 Huang et al., 2017
